# Worldwide perspectives on venom allergy

**DOI:** 10.1016/j.waojou.2019.100067

**Published:** 2019-10-24

**Authors:** Peter Korošec, Thilo Jakob, Harfi Harb, Robert Heddle, Sarah Karabus, Ricardo de Lima Zollner, Julij Selb, Bernard Yu-Hor Thong, Fares Zaitoun, David B.K. Golden, Michael Levin

**Affiliations:** a University Clinic of Respiratory and Allergic Diseases, Golnik, Slovenia; b Department of Dermatoloy and Allergy, University Medical Center Giessen UKGM, Justus-Liebig-University, Giessen, Germany; c National Center of Allergy, Asthma and Immunology, Riyadh, Saudi Arabia; d Royal Adelaide Hospital, Australia; e Laboratory of Translational Immunology, Department of Internal Medicine, School of Medical Sciences, University of Campinas, Brazil; f Department of Rheumatology, Allergy and Immunology, Tan Tock Seng Hospital, Singapore; g Allergy/Immunology, LAUMC, Beirut, Lebanon; h Division of Allergy and Clinical Immunology, Johns Hopkins School of Medicine, Maryland, USA; i Division of Paediatric Allergy, University of Cape Town, South Africa

**Keywords:** Immunotherapy, Venom, Insects, Venom immunotherapy

## Abstract

Venom immunotherapy is the standard of care for people with severe reactions and has been proven to reduce risk of future anaphylactic events. There is a moral imperative to ensure production, supply and worldwide availability of locally relevant, registered, standardized commercial venom extracts for diagnosis and treatment. Insects causing severe immediate allergic reactions vary by region worldwide. The most common culprits include honeybees (*Apis mellifera*), social wasps including yellow jackets (*Vespula* and *Dolichovespula*), paper wasps (*Polistes*) and hornets (*Vespa*), stinging ants (*Solenopsis*, *Myrmecia*, *Pachycondyla*, and *Pogonomyrmex*), and bumblebees (*Bombus*). Insects with importance in specific areas of the world include the Australian tick (*Ixodes holocyclus*), the kissing bug (*Triatoma* spp), horseflies (*Tabanus* spp), and mosquitoes (*Aedes*, *Culex*, *Anopheles*). Reliable access to high quality venom immunotherapy to locally relevant allergens is not available throughout the world. Many current commercially available therapeutic vaccines have deficiencies, are not suitable for, or are unavailable in vast areas of the globe. New products are required to replace products that are unstandardized or inadequate, particularly whole-body extract products. New products are required for insects in which no current treatment options exist. Venom immunotherapy should be promoted throughout the world and the provision thereof be supported by health authorities, regulatory authorities and all sectors of the health care service.

## Introduction

Venom immunotherapy (VIT) remains the definitive treatment for adults and children with insect venom systemic reactions/anaphylaxis. Recent empirical recommendations on optimum treatment duration and modification of maintenance regimes have been published for multiple insects causing venom allergy.^1^, ^2^ However on-going challenges exist with the production, supply and worldwide availability of locally relevant, registered, standardized commercial venom extracts for diagnosis and treatment (see Table 1).

**Table 1:** Availability of allergen extracts regionally^a^. SE: standardized extract, WBE: whole body extract. NA: not available. NR: not relevant in the indicated region.

	Arabian Peninsula^a^	Asia^a^	Australia^a^	Europe^a^	Southern Africa^a^	South America^a^	USA^a^
Honey Bee Venom (*Apis mellifera*)	SE	SE	SE	SE	SE	SE	SE
Wasp Venom Protein (*Polistes* spp)	SE	SE	SE	SE	SE	SE	SE
Yellow Hornet Venom Protein (*Dolichovespula arenaria*)	SE	NR	NR	NR	NR	SE	SE
Yellow Jacket Venom Protein (*Vespula* spp)	SE	SE	SE	SE	SE	SE	SE
Mixed Vespid Venom Protein (mixed yellow jacket, yellow hornet, and white-faced hornet)	SE	SE	NR	NR	NR	NA	SE
Mediterranean wasp (*Polistes dominula*)	SE	SE	NR	SE	NR	NR	NA
Imported black fire ant (*Solensopsis richterii*)	NR	NR	NR	NR	NR	WBE^b^	WBE
Imported red fire ant (*Solenopsis invicta*)	NR	NR	WBE	NR	NR	WBE	WBE
Jack jumper ant (*Myrmecia pilosula*)	NR	NR	SE	NR	NR	NR^b^	NR
Samsum ant (*Pachycondyla senaarensis*)	NA^b^	NA^b^	NR	NR	NR	NA	NR
*Pachycondyla chinensis*	NA^b^	NA^b^	NR	NR	NR	NA	NR
Bumblebee (*Bombus*)	NR	NR	NA	NA	NR	SE^b^	NA
*Polybia paulista*	NR	NR	NR	NR	NR	SE^b^	NR
Australian tick (*Ixodes holocyclus*)	NR	NR	NA	NR	NR	NA	NR
Mosquito (*Aedes aegypt*)	NR	NR	NA	NR	NR	WBE^c^	WBE^b^
Horseflies (*Tabanus* spp)	NR	NR	NR	NR	NR	WBE^c^	WBE^b^
Kissing bug (*Triatoma* spp)	NR	NR	NR	NR	NR	NR	NA

a Availability may differ among countries in each region. The table represents availability in the following countries: Saudi Arabia and Lebanon, Singapore, Australia, Germany, South Africa, Brazil, USA.

b Has been manufactured and used locally but not readily available.

c Available for diagnostic use only

Insects causing severe immediate allergic reactions vary by region worldwide. The most common culprits include honeybees (*Apis mellifera*), social wasps including yellow jackets (*Vespula* and *Dolichovespula*), paper wasps (*Polistes*), hornets (*Vespa*), stinging ants (*Solenopsis*, *Myrmecia*, *Pachycondyla*, and *Pogonomyrmex*), and bumblebees (Bombus). Venom immunotherapy (VIT) is recommended for patients who are IgE sensitized to the culprit insect venom and who have suffered a systemic sting reaction more severe than cutaneous involvement.^1^ The severity cut-off was proposed, because the probability of a more severe reaction after a subsequent sting, if one was stung and reacted with only skin features, is lower than 3%.^1^ However, around 10% of adults with mild systemic reaction have a more severe reaction when re-stung.^3^ On the other hand, roughly 45% of patients with moderate/severe reaction at the initial sting has a less severe reaction on future stings.^3^ The position paper of the European Academy of Allergy and Clinical Immunology (EAACI) recommends venom immunotherapy in adult patients with systemic sting reactions confined to generalized skin symptoms if quality of life is impaired.^2^

Currently, there is no reliable laboratory test or biomarker which can predict the severity of initial, previous, or future allergic sting reactions, and thus the management decision on which patients should be treated with immunotherapy relies on a clinical history of a significant systemic reaction, supported by positive IgE to venom, either by skin testing or sIgE. Measurement of baseline serum tryptase may be of some use in elucidating severe reactors after a field sting. Some studies show only a small proportion (less than 10%) of patients with severe reactions have a baseline serum tryptase above the upper cut-off (11.4 μg/L),^4^ whilst others show 25% of severe reactors have raised tryptase,^5^ predominantly those with mast cell disorders. New methodologies like measurement of sIgE to recombinant venom allergens and/or basophil activation test (BAT) might be helpful. However, recent reports suggest that IgE levels to venom recombinant allergens have no utility in predicting the severity of sting reactions,^6^, ^7^ and BAT testing, which shows promise as a severity predictor in food allergies, has not yet been sufficiently researched for its correlation with severity of sting allergy. Novel biomarkers of severity of allergic reaction to insect sting could significantly improve the care for patients with insect venom allergy, by facilitating better selection of patients in need of VIT.

Recent advances in molecular allergology have allowed a detailed characterization of compositions of commercial venom preparations for immunotherapy.^8^ For efficient treatment all major venom allergens should be present, however recent reports demonstrated that many standardized therapeutic honey bee venom (HBV) preparations are lacking in major allergens including Api m 3, Api m 5 and/or Api m 10.^9^, ^10^ Furthermore, it was demonstrated that predominant Api m 10 sensitization is a risk factor for treatment failure in HBV immunotherapy,^10^ suggesting that underrepresentation of Api m 10 venom allergen in the therapeutic preparation used in this study might significantly affect the efficiency of VIT. Importantly, current standardization of venom allergen extracts, in which manufacturers compare the allergen extract to a reference standard for potency, seems to be insufficient to resolve the issue of missing allergens and thus future standardization should also include molecular allergology approaches and testing.

Unlike honeybee, vespid therapeutic extracts (including preparations of yellow jacket, yellow hornet, white faced hornet and wasp venom) have been demonstrated to contain all major allergens. In the case of yellow jacket, venom is “spiked” with Ves v5 to improve its therapeutic accuracy.^11^ The cross-reactivity of the various *Vespula*, *Dolichovespula*, and *Vespa* species is >90%. In the US, *V.squamosa* was added to the yellow jacket species mix because it is antigenically distinct from other *Vespula*. *Dolichovespula* venom (both *D maculata* and *D arenaria*) is highly cross reactive with *Vespula* and *Vespa*, but it is contained within the mixed vespid extract. It is also available as a single *Dolichovespula arenaria* yellow hornet venom protein extract.

The cross-reactivity of *Vespula* and *Polistes* species is <50%. Various species of *Polistes* cross-react with each other – with the exception of the Mediterranean wasp, *Polistes dominula*, which is prevalent in southern Europe and has invaded much of the United States. The venom of *P dominula* has incomplete cross-reactivity with other *Polistes* species venom; in endemic areas, commercial *Polistes* venom extracts may fail to diagnose or treat patients allergic to *P dominula*.^12^

In some areas, up to 60% of patients who experience an allergic reaction after *Hymenoptera* stings are sensitized to both bee and vespid venom, however the clinical double reactivity to Apidae and Vespidae venom is rare. IgE antibodies against cross-reacting carbohydrate determinants (CCDs), in addition to cross reactivity between homologous venom proteins, are speculated to be a potential cause of clinically irrelevant cross-sensitization. It has been well described that some native *Vespula* and honey bee allergens present the cross-reactive carbohydrate determinants (CCDs) defined by an α1,3-linked fucose residue at the innermost N-acetylglucosamine of the carbohydrate core structure, while *Polistes* and *Polybia* allergens are devoid of CCDs.^13^, ^14^, ^15^

Component resolved diagnostics (CRD) can increase the sensitivity of IgE detection and enable discrimination between true co-sensitivity, primary double sensitivity and cross sensitivity.^16^, ^17^ Although CRD provides greater specificity, there is no consistent evidence of increased sensitivity (compared to existing skin tests and specific IgE tests). CRD thus may increase the clinical utility of IgE testing, but testing is still neither 100% sensitive or specific^7^ even when an extended spectrum of recombinant components is used.^18^ Further research should be focused towards novel tests, like BAT or inclusion of cross-reactive components from vespid venoms.^19^

Protection from sting anaphylaxis is effective as soon as the full dose of VIT is achieved. Sustained unresponsiveness is achieved in the majority of patients after 5 years, and in some patients after 3 years, of maintenance VIT. In studies up to 13 years after completing a course of VIT, 80–90% of unselected patients had no systemic reaction after a sting.^1^ There is evidence that treatment length ≥4 years is better than ≤3 years.^20^, ^21^ The need for indefinite VIT prolongation should be considered in patients with cardiovascular/pulmonary conditions, and according to some authors in patients with a history of severe anaphylaxis or with mast cell disorders.^1^ Currently, there is no biomarker test which would help us to decide whether and when to stop VIT. Notably, it has been shown that neither development of negative skin tests, persistent decline in venom-specific IgE levels, nor induction of blocking IgG antibodies are correlated with tolerance after finishing VIT.^1^

## Distinct regional allergens

### USA

The fire ants (*Solenopsis invicta* and *Solenopsis richteria*), native to South America, are also found in North America,^22^ South America, Australia, New Zealand and several European, Asian, and Caribbean countries where they are often referred to as “imported fire ants”.^23^, ^24^ Local reactions are common, and in areas where imported fire ant is endemic, it represents the major cause of hymenoptera-related hypersensitivity reactions.^25^ The venom comprises both toxic and allergenic components. Sol i1 has phospholipase A1 properties and has some cross reactivity with yellow jacket, honey bee, and wasp phospholipase. Sol i2 and Sol i4 are unique families shared only with other Solenopsis spp. Sol i3 is similar to Ves v5 but has limited cross reactivity.^26^ Whole body extract immunotherapy has been demonstrated to be effective.^27^, ^28^

Bumblebee stings are generally uncommon but are an occupational hazard for greenhouse workers and horticulturalists. Anaphylaxis has been reported in these groups, and VIT has been successful in Europe using locally produced extracts.^29^ There have been rare reports of anaphylaxis to the “sweat bee” of the genus *Halictidae*, but no extracts are available.

There are also some unique species of yellow jacket and *Polistes* in North America. Although some are not fully cross-reactive with the species included in the commercial extracts, none have been added to the commercial products since *V. squamosa* was added to yellow jacket venom mix shortly after regulatory approval almost 40 years ago. There are solitary species of yellow jacket and wasp that are much less widely distributed and much less common culprits in sting anaphylaxis, but there are currently no tests or treatment extracts for specific allergy to these unusual species.

### Brazil

A large number of Hymenoptera are endemic to Brazil and surrounding countries. In comparison to other regions, a wide variety of clinical manifestation related to insect venom has been described for neotropical areas. Brazilian wasp species comprise 33% of the currently identified species worldwide.^30^

*Polistes* is a wasp within the *Polistinae* subfamily of *Vespidae*. *Polistes* contains multiple species which are differentially geographically restricted and show incomplete venom homology.^31^ Other less common members of the *Polistinae* subfamily have also been related to insect-related anaphylaxis.^32^The venom of these *Hymenoptera* is poorly characterized and recombinant allergens from these endemic species are not currently available for diagnosis or treatment.

Within the vespid subfamily falls the *Polybia paulista* species, a neotropical social wasp with a regional distribution in southeast Brazil. In this area, it is of significant clinical importance. Studies have characterized the allergens^33^, ^34^, ^35^, ^36^ but no routine diagnostic or therapeutic extracts are available.

### Australia

Ants are important allergenic insects in Australia.^24^ The predominant insect is jack jumper ant (*Myrmeciapilosula* species complex) and other *Myrmecia* species and the green-head ant (*Rhytidoponera metallica*).^37^

There is a 2–3% risk of severe allergic reaction (anaphylaxis) to jack jumper ant.^38^ Approximately 70% of those that have experienced anaphylaxis to jack jumper ant have anaphylaxis when restung.^38^ Subjects stung previously by the jack jumper ant react commonly (70%) to stings with systemic reactions predominantly involving the cardiovascular and or respiratory systems in addition to any skin reaction.

Immunotherapy for jack jumper ant allergy is available and allergen extracts are well characterized.^39^, ^40^ Immunotherapy is highly effective in prevention of *M pilosula* sting anaphylaxis.^41^ Ultrarush immunotherapy is associated with increased side effects and no improvement in treatment efficacy.^42^ Immunotherapy is not available for the other *Myrmecia* or the *Rhytidoponera* species.

### East Asia and the Arabian Peninsula

Flying stinging hymenoptera of the Apidae and Vespidae families are common in the Middle East and The Arabian Peninsula.^43^ Additional regional stinging insect include *Pachylocondyla* species.

*Pachycondyla chinensis* is a winged ant found predominantly in East Asia^24^, ^44^ and it is a significant risk for anaphylaxis. It is also found as an emerging health problem in the southeastern United States.^45^ *P. sennaarensis* species, commonly known as the "black Samsum ant" is indigenous to Southeast Asia and has been widely reported in the Arabian Peninsula,^26^, ^46^ especially in the Eastern province of Saudi Arabia but not in the North and West.^47^

Studies of cross-reactivity between *Pachycondyla* and imported fire ants are controversial. A Korean study showed no cross-reactivity,^48^ however, another study showed cross-reactivity between *Pachycondyla sennaarensis* and imported fire ant venom by immunoblot testing.^49^

The IgE-binding allergens have been characterized in Samsum venom.^49^ Whole body extracts of *P chinensis*^50^ and *P sennarensis*^51^ have been used as immunotherapy for allergic patients. The Asian hornet, also known as the yellow-legged hornet (*Vespa velutina*), is a species of hornet indigenous to Southeast Asia. It is of concern as an invasive species in some European countries. Specific venom is not available for treatment, although some success has been reported with immunotherapy to yellow jacket venom.^52^

### South Africa

The majority (99%) of fatal insect venom reactions in South Africa occur due to Africanized honebees *Apis mellifera capensis* and *Apis mellifera scutellata,* which are the indigenous species found in this region. Additionally, Africanized honeybees were brought into South America and then migrated into North America, including the southern region of the US. These honeybees are notoriously aggressive in nature. The venom of Africanized honeybee is highly cross-reactive with commonly available honeybee venom used for testing and for VIT.^53^ Hornet and wasp reactions are considerably less common. *Formicidae* (Fire ant, jack jumper ant) have not yet been reported in South Africa.

## Rare allergens (biting insects)

Biting insects can also rarely cause severe reactions.

The Australian tick (*Ixodes holocyclus*) can cause acute anaphylactic reactions^54^ which are generally rare apart from some locations such as north Sydney where it is one of the commoner causes of anaphylaxis. The reaction is not mediated by alpha-gal sensitization but through sensitization to other tick proteins.^55^ No immunotherapy has been established.

The kissing bug (*Triatoma* spp) is an important vector for the transmission of trypanosomiasis. Anaphylactic reactions are reported, predominantly to *Triatoma protracta*, and *Triatoma rubida*.^56^ The major salivary allergen (procalin) is a 20-kDa protein member of the lipocalin family.^57^

Horseflies (*Tabanus* spp) are found all over the world except for some islands and the polar regions. Bites are almost invariably painful and large local reactions are common. Allergic reactions also occur that vary in severity.^58^ Horsefly salivary antigens include Tab a 1, an antigen 5-related protein and Tab a 2, a hyaluronidase.^58^ These antigens show IgE-binding capacity to sera of subjects with wasp sting allergy,^58^ adding credence to the concept of a wasp-horsefly cross reactivity syndrome.^59^

Although local reactions to mosquitoes (*Aedes*, *Culex*, *Anopheles*) are common, anaphylactic reactions are rare.^60^, ^61^ Three recombinant mosquito salivary allergens from *Aedes aegypti* (shared by *Aedes vexans* and other mosquito species) have been cloned and characterized.^62^ This may facilitate the diagnosis of mosquito allergy. Skin testing and immunotherapy is also possible with whole body extracts.^60^ A significant correlation between allergic reactions to *Aedes communis* and bee venom hypersensitivity suggests the occurrence of a “bee-mosquito” cross reactivity syndrome.^63^

## Differences in clinical utility of venom immunotherapy

Hymenoptera venom extracts are widely known to be highly accurate for diagnostic use and remarkably effective for immunotherapy. However, this is not true for all species worldwide. In the controlled clinical trial of VIT in the US, the reported outcome of complete protection in 54/55 patients (98%) who completed the study with sting challenge was based primarily on treatment with mixed vespid venom. VIT with single vespid venoms (yellow jacket or Polistes) may give complete protection in 90–95% of patients, and honeybee VIT is known to give only 80% protection with the 100mcg dose. Studies of treatment failures show that protection can be improved to >90% with 200 mcg maintenance doses of HB venom.^64^

The risk of fire ant anaphylaxis can be substantially reduced by treatment with the available whole body extracts.^27^, ^65^, ^66^ Fire ant venom was shown to have greater potency and activity in skin testing, but there have been no studies of immunotherapy with imported fire ant venom. Interestingly, there have been no reports of treatment failure with imported fire ant whole body extract immunotherapy.

A controlled trial of venom extract immunotherapy for jack jumper ant anaphylaxis showed 98% efficacy, but it also had more adverse reactions than reported for other insect immunotherapy.^41^

A recommended course of VIT is 5 years, however, some authors recommend a shorter duration for lower risk patients and those with a lower severity of initial reaction. Patients with particularly severe initial reactions, those with ongoing exposure (eg, beekeepers, forestry workers) and those with systemic adverse events during immunotherapyare recommended to have an extended course of treatment.^2^ The duration of VIT may also depend on the species. There are insufficient data to make a recommendation for imported fire ant treatment, but the very high attack rate in endemic areas (50% per year) has led to greater caution and extended treatment in patients with imported fire ant anaphylaxis. The duration has not been investigated for jack jumper ant VIT.

## Regional difficulty in access to allergen extracts worldwide

There is concern about the effects of climate change on the species affecting various regions of North America. There has already been documented migration and shifting distribution of some species, and there is likely to be accelerated extinction of species whose habitat is changing irreversibly.^67^ This may bring humans into encounters with species that are not represented in the commercial venom extracts, and some of the species that are currently included may become unavailable due to extinction. Thus, the availability of effective diagnostic and therapeutic venom extracts may be threatened in the near future.

### Shortages or lack of availability

In recent years there have been instances of shortages of venom extract products in many parts of the world, ranging from temporary interruption of a specific product to prolonged interruptions of the supply of multiple products from a single manufacturer or even the complete withdrawal of an entire product line from a large region of the world. In such situations, there is a need for patients to be treated with venoms from a different supplier. Unfortunately, in countries with a single supplier, these situations have led to complete lack of availability for long periods of time, in which case patients cannot begin or continue VIT regardless of the danger of life-threatening anaphylaxis. This occurred in Canada, Australia, Southeast Asia and South Africa, and there is now a very real danger of the same thing happening in the US.

During times of shortage there has been some expert opinion offered to help guide the clinician and the patients.^68^ Based on limited evidence and much experience, the venom shortage guidelines suggested that greater discretion should be applied in prescribing VIT, with reassurance (rather than VIT) for patients with milder conditions, and that treatment could be discontinued in those who had a history of only dermal reactions.^68^ It was also suggested that the number of venoms used could be limited in many cases, and that maintenance intervals could be safely extended by 25%–50%.^68^ There was less enthusiasm for reducing the dose to 50 mcg in adults, but this appears to be quite safe in children. There was also discussion of recommending discontinuation of VIT in patients who had remained on treatment longer than 5 years for quality of life reasons rather than medical necessity (high-risk patients). One of the lessons learned from these times of shortages suggest that there is some interchangeability of venom preparations and dose regimes.^69^

Even more concerning is the lack of registration of products in large areas of the globe where such products may be indicated, and the lack of availability of standardized extracts for several important venoms.

### Major unmet needs

Venom immunotherapy is the standard of care for people with severe reactions and has been proven to reduce risk of future anaphylactic events. However, many products are unavailable in vast areas of the world and are not funded by public health services. There is a moral imperative to ensure production, supply and worldwide availability of locally relevant, registered, standardized commercial venom extracts for diagnosis and treatment. In addition, non-specific treatment (e.g. self-medication with epinephrine autoinjectors) is lacking in many areas of the world, particularly in lower- and middle-income countries and rural areas.^70^

There is a need for new products for venoms of importance in disparate regions of the world, and for the promotion of such products that are available to be improved, registered and made available to those who could benefit. Monopolies of single manufacturers making a specific immunotherapy product is potentially problematic for commercial reasons and for ensuring an uninterrupted supply chain.

It is important that patients who are sensitized to minor honeybee venom allergens are treated with HBV preparations which contain the full spectrum of HBV allergens.

Unmet needs include venom-based immunotherapy for imported fire ant venom, more potent (eg, adjuvanted) venom products and immunotherapy to the venoms of additional species (including bumblebee and *Polybia paulista*). It is also important that immunotherapy products of species-specific venoms such as Polistes that differ in varied parts of the globe reflect the venoms in the local population and are either produced specifically for those areas or clearly marked with the species concerned and an explanation that it may not cover all allergens in some parts of the globe.

New procedures for venom extraction from *Solenopsis* species have been studied using insect stress and dual phase mixture of apolar organic solvent and water. However, its application for diagnosis and venom immunotherapy needs more studies.^71^

Reagents for skin or intradermal testing for stinging insect hypersensitivity are not available in many regions of the world making diagnosis reliant on clinical history coupled with IgE testing.

Epidemiological data on insect envenomation, including *Hymenoptera*, spider, scorpion and snake, are lacking. There is an urgent need for surveillance to identify changes in the presence and population of insect species in all affected regions, and to document adverse reactions to these species to identify species for which new products or replacement products must be developed. Epidemiological data for these neglected public health issues in developing countries is required to convince public health authorities and companies to invest in diagnostic and treatment.

Regulatory issues in various parts of the world are a significant obstacle to the fulfilling these needs. The need for wider availability of venom immunotherapy should be driven by allergists, and also should be encouraged by primary health care providers, who need to be made aware of the availability, efficacy, and safety of VIT.

Currently, regulatory approval for new venom products in many parts of the world requires standard clinical trials with sting challenges to establish efficacy, and the submission of local dossiers to each country considering the regulation of such products. Possible solutions for this include allowing registering products that have been approved in other parts of the world to be registered elsewhere based on data showing the same species of insects and venoms causing the local allergy, the registration of new well-characterized venom products with limited clinical studies and without sting challenge, based on the concept of “bio-similars” and the use of surrogate markers for efficacy. The authors strongly recommend that governments worldwide consider venom allergy as “orphan diseases” in order to facilitate the development, registration and availability of venom products.

## Conclusion and recommendations

Reliable access to high quality venom immunotherapy to locally relevant allergens is not available throughout the world. Many current commercially available therapeutic vaccines have deficiencies, are not suitable for, or are unavailable in vast areas of the globe. New products are required to replace products that are unstandardized or inadequate, particularly whole-body extract products. New products are required for insects in which no current treatment options exist. Venom immunotherapy should be promoted throughout the world and the provision thereof be supported by health authorities, regulatory authorities and all sectors of the health care service.

## References

[1] Golden D., Demain J., Freeman T. (2017). Stinging insect hypersensitivity: a practice parameter update 2016. Ann Allergy Asthma Immunol.

[2] Sturm G.J., Varga E.M., Roberts G. (2018 Apr). EAACI guidelines on allergen immunotherapy: hymenoptera venom allergy. Allergy Eur J Allergy Clin Immunol.

[3] Reisman R.E. (1992 Sep). Natural history of insect sting allergy: relationship of severity of symptoms of initial sting anaphylaxis to re-sting reactions. J Allergy Clin Immunol.

[4] Ruëff F., Przybilla B., Biló M.B. (2009 Nov). Predictors of severe systemic anaphylactic reactions in patients with hymenoptera venom allergy: importance of baseline serum tryptase-a study of the european academy of allergology and clinical immunology interest group on insect venom hypersensitivity. J Allergy Clin Immunol.

[5] Bonadonna P., Perbellini O., Passalacqua G. (2009 Mar). Clonal mast cell disorders in patients with systemic reactions to Hymenoptera stings and increased serum tryptase levels. J Allergy Clin Immunol.

[6] Šelb J., Kogovšek R., Šilar M., Košnik M., Korošec P. (2016 Apr). Improved recombinant Api m 1- and Ves v 5-based IgE testing to dissect bee and yellow jacket allergy and their correlation with the severity of the sting reaction. Clin Exp Allergy.

[7] Šelb J., Bidovec Stojković U., Bajrović N. (2018 Nov - Dec). Limited ability of recombinant Hymenoptera venom allergens to resolve IgE double sensitization. J Allergy Clin Immunol Pract.

[8] Spillner E., Blank S., Jakob T. (2014 Feb 28). Hymenoptera allergens: from venom to “venome. Front Immunol.

[9] Frick M., Fischer J., Helbling A. (2016 Dec). Predominant Api m 10 sensitization as risk factor for treatment failure in honey bee venom immunotherapy. J Allergy Clin Immunol.

[10] Blank S., Etzold S., Darsow U. (2017 Oct 3). Component-resolved evaluation of the content of major allergens in therapeutic extracts for specific immunotherapy of honeybee venom allergy. Hum Vaccines Immunother.

[11] Vos B., Köhler J., Müller S., Stretz E., Ruëff F., Jakob T. (2013 Apr). Spiking venom with rVes v 5 improves sensitivity of IgE detection in patients with allergy to Vespula venom. J Allergy Clin Immunol.

[12] Blanca M., Garcia F., Miranda A. (1991 Feb). Determination of IgE antibodies to Polistes dominulus, Vespula germanica and Vespa crabro in sera of patients allergic to vespids. Allergy.

[13] Ollert M., Blank S. (2015). Anaphylaxis to Insect Venom Allergens : Role of Molecular Diagnostics.

[14] Blank S., Neu C., Hasche D., Bantleon F.I., Jakob T., Spillner E. (2013 Apr). Polistes species venom is devoid of carbohydrate-based cross-reactivity and allows interference-free diagnostics. J Allergy Clin Immunol.

[15] Perez-Riverol A., Miehe M., Jabs F. (2018 May). Venoms of Neotropical wasps lack cross-reactive carbohydrate determinants enabling reliable protein-based specific IgE determination. J Allergy Clin Immunol.

[16] Müller U.R., Johansen N., Petersen A.B., Fromberg-Nielsen J., Haeberli G. (2009 Apr). Hymenoptera venom allergy: analysis of double positivity to honey bee and Vespula venom by estimation of IgE antibodies to species-specific major allergens Api m1 and Ves v5. Allergy.

[17] Jakob T., Müller U., Helbling A., Spillner E. (2017 Oct). Component resolved diagnostics for hymenoptera venom allergy. Curr Opin Allergy Clin Immunol.

[18] Köhler J., Blank S., Müller S. (2014 May). Component resolution reveals additional major allergens in patients with honeybee venom allergy. J Allergy Clin Immunol.

[19] Jakob T., Rafei-Shamsabadi D., Spillner E., Müller S. (2017). Diagnostics in Hymenoptera venom allergy: current concepts and developments with special focus on molecular allergy diagnostics. Allergo J Int.

[20] Keating M.U., Kagey-Sobotka A., Hamilton R.G., Yunginger J.W. (1991 Sep). Clinical and immunologic follow-up of patients who stop venom immunotherapy. J Allergy Clin Immunol.

[21] Lerch E., Müller U.R. (1998 May). Long-term protection after stopping venom immunotherapy: results of re-stings in 200 patients. J Allergy Clin Immunol.

[22] Kemp S.F., deShazo R.D., Moffitt J.E., Williams D.F., Buhner W.A. (2000 Apr). Expanding habitat of the imported fire ant (Solenopsis invicta): a public health concern. J Allergy Clin Immunol.

[23] Tankersley M.S. (2008 Aug). The stinging impact of the imported fire ant. Curr Opin Allergy Clin Immunol.

[24] Srisong H., Daduang S., Lopata A.L. (2016 Jan). Current advances in ant venom proteins causing hypersensitivity reactions in the Asia-Pacific region. Mol Immunol.

[25] Steigelman D.A., Freeman T.M. (2013 Oct). Imported fire ant allergy: case presentation and review of incidence, prevalence, diagnosis, and current treatment. Ann Allergy Asthma Immunol.

[26] Hoffman D.R., Dove D.E., Jacobson R.S. (1988 Nov). Allergens in Hymenoptera venom. XX. Isolation of four allergens from imported fire ant (Solenopsis invicta) venom. J Allergy Clin Immunol.

[27] Freeman T.M., Hylander R., Ortiz A., Martin M.E. (1992 Aug). Imported fire ant immunotherapy: effectiveness of whole body extracts. J Allergy Clin Immunol.

[28] Tankersley M.S., Walker R.L., Butler W.K. (2002 Aug). Safety and efficacy of an imported fire ant rush immunotherapy protocol with and without prophylactic treatment. J Allergy Clin Immunol.

[29] De Jong N.W., Vermeulen A.M., De Groot H. (1999 Sep). Allergy to bumblebee venom. III. Immunotherapy follow-up study (safety and efficacy) in patients with occupational bumblebee-venom anaphylaxis. Allergy.

[30] Locher G., Togni O., Silveira O., Giannotti E. (2014). The social wasp fauna of a riparian forest in southeastern Brazil (hymenoptera, Vespidae). Sociobiology.

[31] Severino M.G., Campi P., Macchia D. (2006 Jul). European Polistes venom allergy. Allergy.

[32] Guimaraes M. (2008). Death Swarm. Pesqui FAPESP [Internet]. https://revistapesquisa.fapesp.br/en/2008/11/01/death-swarm-2/.

[33] dos Santos L.D., Santos K.S., Pinto J.R.A. (2010 Aug). Profiling the proteome of the venom from the social wasp Polybia paulista: a clue to understand the envenoming mechanism. J Proteome Res.

[34] Justo Jacomini D.L., Gomes Moreira S.M., Campos Pereira F.D., Zollner R. de L., Brochetto Braga M.R. (2014 May). Reactivity of IgE to the allergen hyaluronidase from polybia paulista (hymenoptera, Vespidae) venom. Toxicon.

[35] Perez-Riverol A., Campos Pereira F.D., Musacchio Lasa A. (2016 Dec). Molecular cloning, expression and IgE-immunoreactivity of phospholipase A1, a major allergen from Polybia paulista (Hymenoptera: Vespidae) venom. Toxicon.

[36] Perez-Riverol A., Campos Pereira F.D., Musacchio Lasa A. (2013 Dec). Profiling the proteome of the venom from the social wasp polybia paulista: a clue to understand the envenoming mechanism. Toxicon [Internet].

[37] Brown S.G.A., van Eeden P., Wiese M.D. (2011 Jul). Causes of ant sting anaphylaxis in Australia: the Australian ant venom allergy study. Med J Aust.

[38] Brown S.G.A., Franks R.W., Baldo B.A., Heddle R.J. (2003 Jan). Prevalence, severity, and natural history of jack jumper ant venom allergy in Tasmania. J Allergy Clin Immunol.

[39] Wiese M.D., Davies N.W., Chataway T.K., Milne R.W., Brown S.G.A., Heddle R.J. (2011 Jan). Stability of Myrmecia pilosula (jack jumper) ant venom for use in immunotherapy. J Pharm Biomed Anal.

[40] Wanandy T., Wilson R., Gell D. (2018 Sep). Towards complete identification of allergens in Jack Jumper (Myrmecia pilosula) ant venom and their clinical relevance: an immunoproteomic approach. Clin Exp Allergy.

[41] Brown S.G.A., Wiese M.D., Blackman K.E., Heddle R.J. (2003 Mar). Ant venom immunotherapy: a double-blind, placebo-controlled, crossover trial. Lancet (London, England).

[42] Brown S.G.A., Wiese M.D., van Eeden P. (2012 Jul). Ultrarush versus semirush initiation of insect venom immunotherapy: a randomized controlled trial. J Allergy Clin Immunol.

[43] Khoobdel M., Tavassoli M., Salari M., Firozi F. (2014). The Stinging Apidae and Vespidae (Hymenoptera: Apocrita) in Iranian Islands, Qeshm, Abu–Musa, Great Tunb and Lesser Tunb on the Persian Gulf.

[44] Shek L.P.-C., Ngiam N.S.P., Lee B.-W. (2004 Aug). Ant allergy in Asia and Australia. Curr Opin Allergy Clin Immunol.

[45] Nelder M.P., Paysen E.S., Zungoli P.A., Benson E.P. (2006 Sep). Emergence of the introduced ant Pachycondyla chinensis (Formicidae: ponerinae) as a public health threat in the southeastern United States. J Med Entomol.

[46] Alsharani M., Alanazi M., Alsalamah M. (2009). Black ant stings caused by Pachycondyla sennaarensis: a significant health hazard. Ann Saudi Med.

[47] Al-Khalifa M.S., Mashaly A.M.A., Siddiqui M.I., Al-Mekhlafi F.A. (2015 Sep). Samsum ant, Brachyponera sennaarensis (formicidae: ponerinae): distribution and abundance in Saudi Arabia. Saudi J Biol Sci.

[48] Yun Y.Y., Ko S.H., Park J.W., Hong C.S. (1999 Oct). Anaphylaxis to venom of the Pachycondyla species ant. J Allergy Clin Immunol.

[49] Reunala T, Brummer-Korvenkontio H, Saarinen K, Rosanen G, Lestringant D, Hoffman DR. Characterization of IgE-binding allergens in Samsum ant venom. In: Journal of Allergy and Clinical Immunology. p. S108.

[50] Kim S.S., Park H.S., Kim H.Y., Lee S.K., Nahm D.H. (2001 Jun). Anaphylaxis caused by the new ant, Pachycondyla chinensis: demonstration of specific IgE and IgE-binding components. J Allergy Clin Immunol.

[51] Dib G., Guerin B., Banks W.A., Leynadier F. (1995 Oct). Systemic reactions to the Samsum ant: an IgE-mediated hypersensitivity. J Allergy Clin Immunol.

[52] Goldberg A., Shefler I., Panasoff J., Paitan Y., Confino-Cohen R. (2013). Immunotherapy with commercial venoms is efficacious for anaphylactic reactions to Vespa orientalis stings. Int Arch Allergy Immunol.

[53] Schumacher M.J., Schmidt J.O., Egen N.B., Dillon K.A. (1992 Jul). Biochemical variability of venoms from individual European and Africanized honeybees (Apis mellifera). J Allergy Clin Immunol.

[54] van Nunen S. (2015). Tick-induced allergies: mammalian meat allergy, tick anaphylaxis and their significance. Asia Pac Allergy [Internet].

[55] Mateos-Hernández L., Villar M., Moral A. (2017). Tick-host conflict: immunoglobulin E antibodies to tick proteins in patients with anaphylaxis to tick bite. Oncotarget [Internet].

[56] Moffitt J.E., Venarske D., Goddard J., Yates A.B., deShazo R.D. (2003 Aug). Allergic reactions to Triatoma bites. Ann Allergy Asthma Immunol.

[57] Paddock C.D., McKerrow J.H., Hansell E., Foreman K.W., Hsieh I., Marshall N. (2001 Sep). Identification, cloning, and recombinant expression of procalin, a major triatomine allergen. J Immunol.

[58] Ma D., Li Y., Dong J. (2011 Jan). Purification and characterization of two new allergens from the salivary glands of the horsefly. Tabanus yao. Allergy..

[59] Quercia O., Emiliani F., Foschi F.G., Stefanini G.F. (2008 Jun). The wasp-horsefly syndrome. Eur Ann Allergy Clin Immunol.

[60] McCormack D.R., Salata K.F., Hershey J.N., Carpenter G.B., Engler R.J. (1995 Jan). Mosquito bite anaphylaxis: immunotherapy with whole body extracts. Ann Allergy Asthma Immunol.

[61] Reiter N., Reiter M., Altrichter S. (2013 Oct). Anaphylaxis caused by mosquito allergy in systemic mastocytosis. Lancet (London, England).

[62] Simons F.E., Peng Z. (2001). Mosquito allergy: recombinant mosquito salivary antigens for new diagnostic tests. Int Arch Allergy Immunol.

[63] Scala E., Pirrotta L., Uasuf C.G. (2018). Aedes communis reactivity is associated with Bee venom hypersensitivity: an in vitro and in vivo study. Int Arch Allergy Immunol.

[64] Rueff F., Vos B., Oude Elberink J. (2014). Predictors of clinical effectiveness of Hymenoptera venom immunotherapy. Clin Exp Allergy.

[65] Arseneau A.M., Nesselroad T.D., Dietrich J.J. (2013 Dec). A 1-day imported fire ant rush immunotherapy schedule with and without premedication. Ann Allergy Asthma Immunol.

[66] Adams K.E., Johnson K.S. (2019 May 1). Safety of repeated imported fire ant ultra-rush protocols. Mil Med.

[67] Demain J.G., Gessner B.D., McLaughlin J.B., Sikes D.S., Foote J.T. (2009). Increasing insect reactions in Alaska: is this related to changing climate?. Allergy Asthma Proc.

[68] Golden D.B.K., Bernstein D.I., Freeman T.M., Tracy J.M., Lang D.M., Nicklas R.A. (2017 Mar - Apr). AAAAI/ACAAI joint venom extract shortage task force report. J Allergy Clin Immunol Pract.

[69] Stoevesandt J., Trautmann A. (2019). Lessons from Times of Shortage: Interchangeability of Venom Preparations and Dosing Protocols.

[70] Simons F.E.R. (2010 May). World Allergy Organization survey on global availability of essentials for the assessment and management of anaphylaxis by allergy-immunology specialists in health care settings. Ann Allergy Asthma Immunol.

[71] Goncalves Paterson Fox E., Russ Solis D., Delazari Dos Santos L. (2013 Apr). A simple, rapid method for the extraction of whole fire ant venom (Insecta: formicidae: Solenopsis). Toxicon.

